# *MUC1* is associated with *TFF2* methylation in gastric cancer

**DOI:** 10.1186/s13148-020-00832-6

**Published:** 2020-03-02

**Authors:** Yuqiu Ge, Gaoxiang Ma, Hanting Liu, Yadi Lin, Gang Zhang, Mulong Du, Meilin Wang, Haiyan Chu, Haiyan Zhang, Zhengdong Zhang

**Affiliations:** 1 Department of Environmental Genomics, School of Public Health, Jiangsu Key Laboratory of Cancer Biomarkers, Prevention and Treatment, Collaborative Innovation Center For Cancer Personalized Medicine, Nanjing Medical University, 101 Longmian Avenue, Jiangning District, Nanjing, 211166 China; 2 Department of Genetic Toxicology, The Key Laboratory of Modern Toxicology of Ministry of Education, School of Public Health, Nanjing Medical University, Nanjing, China; 3 Department of Public Health and Preventive Medicine, Wuxi School of Medicine, Jiangnan University, Wuxi, China; 4 State Key Laboratory of Natural Medicines, School of Traditional Chinese Pharmacy, China Pharmaceutical University, Nanjing, China; 5 Department of Neurology, Children’s Hospital of Nanjing Medical University, Nanjing, China; 6 Department of Gastroenterology, Xuzhou Clinical College of Nanjing Medical University, Xuzhou Central Hospital, 199 Jiefang South Road, Xuzhou, 221009 China

**Keywords:** *MUC1*, *TFF2*, Methylation, Gastric cancer, Molecular epidemiology

## Abstract

**Background:**

Emerging evidence has shown that *MUC1* and *TFF2* play crucial roles in the *H. pylori*-infected pathogenesis of gastric cancer (GC). A recent study revealed that *H. pylori* infection induced obviously increased *Tff2* methylation levels in *Muc1*^−/−^ mice compared with controls. However, little is known of the molecular mechanism on *MUC1* regulating the expression of *TFF2*.

**Methods:**

We conducted a correlation analysis of *MUC1* and *TFF2* in public databases and our adjacent GC tissues. Besides, *MUC1* overexpression vector or small interfering RNA (siRNA) was transfected into GC cells to assess the change in *TFF2* expression. Furthermore, the methylation status of *TFF2* was measured by bisulfite sequencing PCR (BSP).

**Results:**

The expression of *MUC1* was significantly lower in non-cardia and cardia tumor tissues than that in normal tissues. Downregulation of *TFF2* expression was also observed in GC tissues. In addition, we found that *MUC1* expression was positively associated with *TFF2* expression in GC tissues, especially among GC patients with *H. pylori* infection. Overexpression of *MUC1* in BGC-823 and SGC-7901 cell lines substantially increased the *TFF2* expression, whereas knockdown of *MUC1* reverted this effect. Moreover, *MUC1* was negatively related to the methylation of *TFF2* in the co-expression analysis. The results of BSP experiments showed that compared with negative vector group, the methylation level of *TFF2* was decreased in GC cells transfected with *MUC1* overexpression vector. Additionally, survival analysis indicated that GC patients with lower level of *MUC1* or *TFF2* had a worse outcome.

**Conclusion:**

Our results indicated that *MUC1* was associated with the methylation of *TFF2*, which may have implications for *TFF2* expression in GC. These findings warrant further research toward the underlying mechanism of *MUC1* influenced the *TFF2* methylation.

## Background

Although the incidence and mortality rates of gastric cancer (GC) are decreased over the past few decades, GC is still a severe public health problem. Reportedly, GC is the third leading cause of cancer death with the highest incidence occurring in Eastern Asia [1, 2]. GC is a complex disease, arising from the interaction of environmental and genetic factors. It has been established that *H. pylori* infection is the most important risk factor for GC [3]. Accumulating evidence has shown that eradication of *H. pylori* infection was significantly associated with the reduced incidence of GC, highlighting the pathogenic role of *H. pylori* in GC [4, 5].

The tumorigenesis progression of GC is involved in multiple stages and develops from normal epithelia, chronic gastritis, atrophic gastritis, intestinal metaplasia, and dysplasia to carcinoma [6, 7]. The original progression of GC is gastric mucosa lesions. Thus, it is necessary to maintain the function of gastric mucosa, which prevents infection by the variety of microorganisms. Mucin glycoproteins are the key components of the mucosal barriers [8]. Mucin 1 (*MUC1*) is a kind of cell surface mucins and is abundant in mucosal tissues, especially in normal gastric epithelial cells [9]. It has been reported that *MUC1* played a crucial role in *H. pylori*-associated gastritis through acting as a protective physical barrier against pathogens [8, 10]. Previous study has shown that colonization of *H. pylori* in *MUC1*-deficient (*Muc1*^−/−^) mice was remarkably higher than that in *Muc1*^+/+^ mice. Besides, *MUC1* was also implicated in decreasing the inflammation induced by *H. pylori* [11]. In addition, it is noteworthy that previous genome-wide association studies (GWASs) have identified several risk loci located in *MUC1* gene, which were significantly associated with the susceptibility of GC [12, 13].

Trefoil factor family 2 (*TFF2*), a member of secreted peptides, is also expressed in gastric mucosa and triggers cell migration signaling to promote epithelial repair [14, 15]. It has been documented that *H. pylori* infection contributes to methylation and silencing of *TFF2*, thus involving in GC development [16]. Garrett et al. demonstrated that MUC1 acted as a negative regulator of the NLRP3 inflammasome activation and protected against *H. pylori* pathogenesis. In addition, interleukin (IL)-1 promoted precancerous progression in *Muc1*^−/−^ mice through regulating the methylation status and expression of *Tff2* [17]. However, the molecular mechanism of *MUC1* involved in the regulation of *TFF2* is largely unexplored. Therefore, we hypothesized that the aberrant *MUC1* regulates the methylation status of *TFF2*, thus contributes to silence of *TFF2* in the carcinogenesis of GC. In the present study, we demonstrated that both *MUC1* and *TFF2* were downregulated in GC tissues. Overexpression of *MUC1* was significantly associated with decreased the *TFF2* methylation and increased the *TFF2* expression.

## Materials and methods

### Samples and datasets

This study was approved by the international review board of Nanjing Medical University. A total of 59 GC patients were recruited from the Second Affiliated Hospital of Nanjing Medical University. We detected the expression of *MUC1* and *TFF2* in adjacent GC tissues and compared the difference of expression between *H. pylori* infected (*n* = 31) and non-infected (*n* = 28) GC patients. The RNA-seq data from The Cancer Genome Atlas (TCGA) database was acquired by the UCSC Xena project (http://xena.ucsc.edu/). In this study, we downloaded gene expression (*n* = 417, IlluminaHiSeq RNASeq platform) and DNA methylation (*n* = 398, Illumina Infinium HumanMethylation450 platform) profiles. Samples with both gene and methylation expression profiles were included in the further correlation analysis (*n* = 373). Besides, we analyzed gene expression array deposited in Gene Expression Omnibus (GEO, GSE29272), which has the global gene expression of 72 gastric non-cardia tumor and 62 gastric cardia tumor and their paired normal tissues [18]. We also applied a GEO dataset (GSE74577), which was conducted to investigate the gene expression changes in response to *H. pylori* infection [19]. Additionally, GSE99553 was used to compare methylation level of CpG sites between GC patients with *H. pylori* infection (*n* = 28) and non-infected patients (*n* = 28).

### Cell culture and transfection

The full-length *MUC1* cDNA was synthesized and constructed into the pEGFP-N1 vector. GC cell lines (BGC-823 and SGC-7901) were transfected with overexpression vector using Lipofectamine 2000 reagent (Invitrogen, Carlsbad, CA, USA). Vectors in this study were confirmed by DNA sequencing. Small interfering RNA (siRNA) was synthesized to inhibit *MUC1* expression. The sequence of siRNA is the following: CGGGATACCTACCATCCTA.

### Quantitative real-time PCR (qRT-PCR) analysis

Total RNA from BGC-823 and SGC-7901 cell lines was isolated by Trizol Reagent (Invitrogen, CA, USA), and cDNA was synthesized by Primescript RT Reagent Kit (TaKaRa, Osaka, Japan). The real-time PCR was conducted with ABI 7900HT Real-Time PCR System (Applied Biosystem, Foster City, CA, USA) using SYBR Green assays. *GAPDH* was treated as internal control to quantify the expression levels of *TFF2* and *MUC1* by 2^-Δct^ method. The primer sequences are as follows: F: 5′-GCTGTTTCGACTCCAGTGTCA-3′ and R: 5′-CCACAGTTTCTTCGGTCTGAG-3′ for *TFF2*, F: 5′-CCTACCATCCTATGAGCGAGTAC-3′ and R: 5′-GCTGGGTTTGTGTAAGAGAGGC-3′ for *MUC1*, F: 5′-CCGGGAAACTGTGGCGTGATGG-3′ and R: 5′-AGGTGGAGGAGTGGGTGTCGCTGTT-3′ for *GAPDH*.

### Bisulfite sequencing PCR (BSP)

DNA was isolated from BGC-823 and SGC-7901 cell lines by DNeasy Blood & Tissue Kit (QIAGEN, Hilden, Germany) according to the manufacturer’s protocols. We used EpiTect Fast DNA Bisulfite Kit (QIAGEN) to accomplish bisulfite conversion of genomic DNA. The primers for amplification are shown in Supplementary Table 1. Then, the PCR products were purified and cloned into pTG19-T Vector (Generay, Co., Ltd., Shanghai, China). Ten subclones from each cell line with two replicated experiments were chosen for sequence. Methylation level was calculated as the number of CpG methylated loci divided by the number of detected CpG loci.

### Statistical analysis

Student’s *t* test or paired *t* test was used to compare the differences of gene expression or methylation level. Associations of gene expression and methylation level of CpG sites were assessed by Pearson’s correlation analysis. We applied an online tool, Kaplan-Meier Plotter, to calculate hazard ratios (HRs) and their correspondence 95% confidence interval (95% CI) [20]. The log-rank test was applied to assess the significant effect of gene expression level on survival of patients. We used multivariate cox regression analysis to assess the prognostic value of *MUC1* and *TFF2*, with adjustment for age, sex, grade, and stage of GC patients. All statistical analyses were conducted by the R 3.5.1 software, and two-tailed *P* value < 0.05 was considered as statistically significant.

## Results

### Downregulation of *MUC1* and *TFF2* in GC

**Fig. 1:**
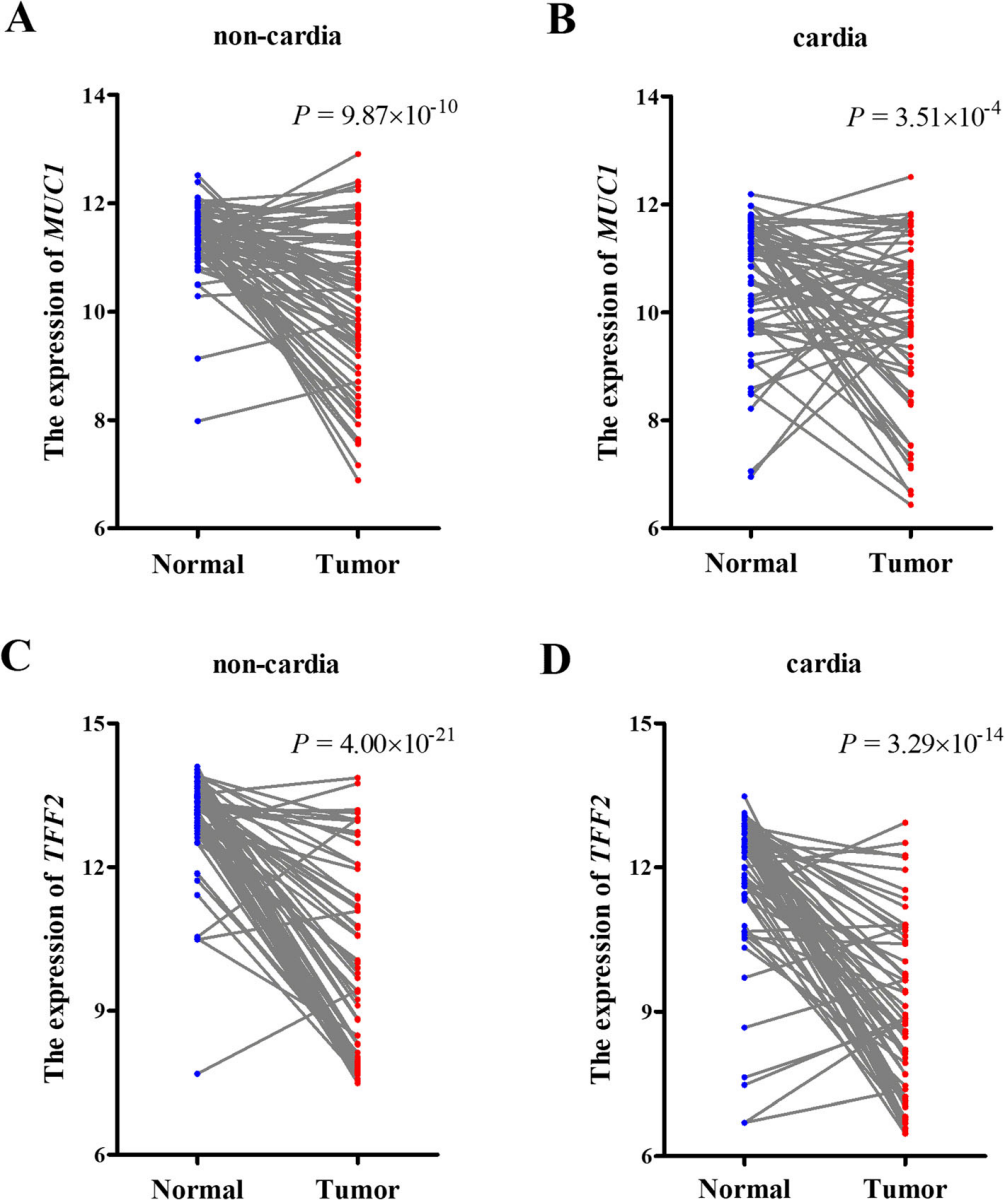
Downregulation of *MUC1* and *TFF2* in GC tissues. The expression level of *MUC1* was significantly lower in non-cardia (**a**) and cardia (**b**) tumor tissues than that in adjacent normal tissues. **c**, **d** Downregulation of *TFF2* expression in non-cardia (**c**) and cardia (**d**) tumor tissues. All *P* values were from paired *t* tests

We assessed the expression level of *MUC1* and *TFF2* in gastric non-cardia and cardia tissues and their correspondence normal tissues from GEO database (GSE29272). As shown in Fig. 1, the expression of *MUC1* in non-cardia tumor tissue was significantly lower than that in paired adjacent normal tissues (*P* = 9.87 × 10^− 10^; Fig. 1a). Additionally, compared with normal tissues, a similar downregulated expression of *MUC1* was observed in the cardia tumor tissue (*P* = 3.51 × 10^− 4^; Fig. 1b). As with *MUC1*, *TFF2* was significantly decreased in non-cardia and cardia tumor tissues compared with that in adjacent normal tissues (*P* = 4.00 × 10^− 21^ and *P* = 3.29 × 10^− 14^, respectively; Fig. 1c, d). Both *MUC1* and *TFF2* expression showed a slight decreased in cardia tissue compared with non-cardia tumor tissue, but there were no significantly differences (Supplementary Figure 1).

Additionally, we detected the expression of *MUC1* and *TFF2* in adjacent GC tissues from our cohort. Compared with non-infected patient, *MUC1* and *TFF2* expression were not statistically significant and showed a slightly decreased trend in *H. pylori*-infected patients (Supplementary Figure 2A-B). The results of GEO dataset (GSE74577) indicated that the expression of *MUC1* but not *TFF2* was significantly decreased in GES-1 cells after 24-h infection with *H. pylori* (*P* = 4.49 × 10^− 4^, *P* = 0.582, respectively; Supplementary Figure 2C-D). Besides, we found the methylation level of CpG sites (cg13804478, cg18804777, and cg24512973) located in *MUC1* were significantly higher in *H. pylori*-infected patients than in non-infected patient (Supplementary Figure 3). For *TFF2*, there was one CpG site; cg26403416 was significantly increased in *H. pylori*-infected patients than in non-infected patients in both GSE99553 and TCGA datasets (data was not shown).

### *MUC1* positively regulates the expression of *TFF2*

**Fig. 2:**
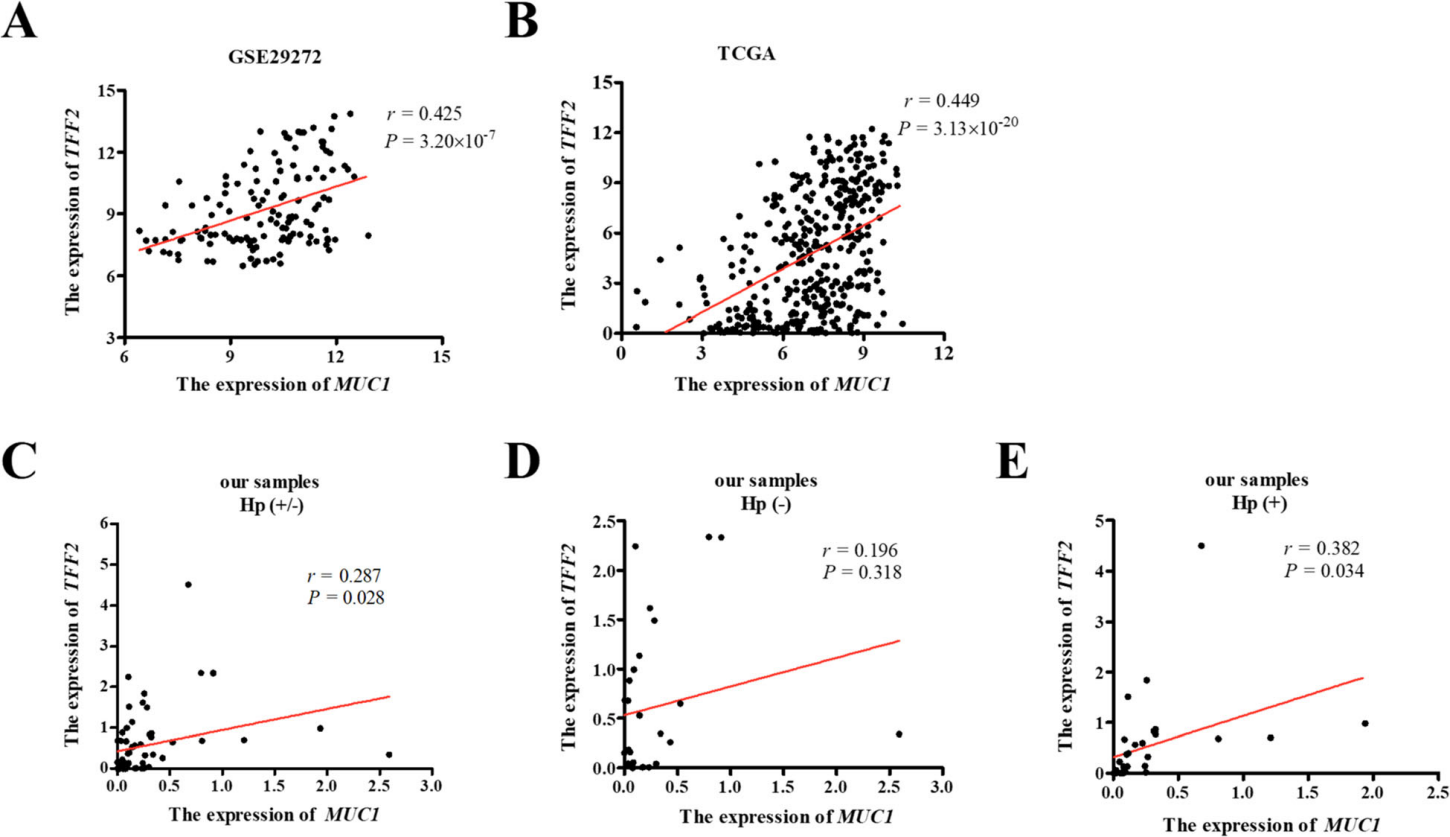
Correlations between *MUC1* and *TFF2* expression level. **a**, **b** Correlation analysis between *MUC1* and *TFF2* expression in GSE29272 (**a**) and TCGA (**b**) databases and in our samples according to *H. pylori* infection status (**c** total samples; **d** GC patient without *H. pylori* infection; **e** GC patient with *H. pylori* infection). The *r* values and *P* values were from Pearson’s correlation analysis

In the present study, we conducted the co-expression between *MUC1* and *TFF2* in the GSE29272 database. The correlation analysis results showed that *MUC1* expression was positively associated with *TFF2* expression in GC tissues (*r* = 0.425, *P* = 3.20 × 10^− 7^; Fig. 2a). The similar positive relationship between *MUC1* and *TFF2* was found in the TCGA database (*r* = 0.449, *P* = 3.13 × 10^− 20^; Fig. 2b). In addition, we investigate the relationship between the expression of *MUC1* and *TFF2* in our cohort. The result demonstrated significantly positive correlation of *MUC1* and *TFF2* expression among GC patients with *H. pylori* infection but not for those without infection (total: *r* = 0.287, *P* = 0.028; *H. pylori* negative: *r* = 0.196, *P* = 0.318; *H. pylori* positive: *r* = 0.382, *P* = 0.034; Fig. 2c–e). Furthermore, the positive correlations between the expression of *MUC1* and *TFF2* were also observed in other clinical features, including different age, sex, stage, and grade of GC patients (Supplementary Table 2).

**Fig. 3:**
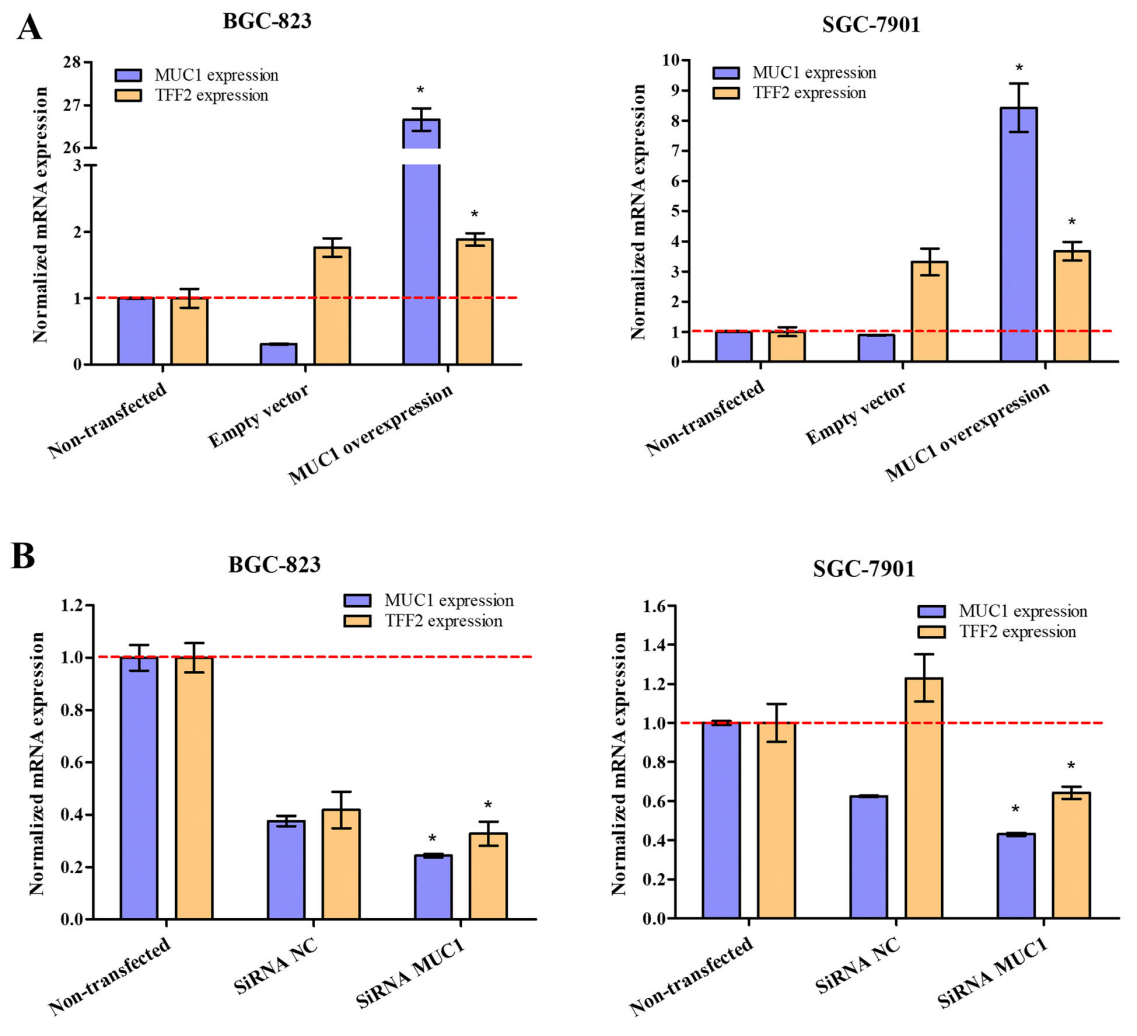
Effect of *MUC1* overexpression or inhibition on the *TFF2* expression. **a** BGC-823 and SGC-7901 cell lines were treated with non-transfection, transfected with empty vector or overexpression vector of *MUC1*. **b** BGC-823 and SGC-7901 cell lines were treated with non-transfection, transfected with negative control siRNA (siRNA NC) and siRNA *MUC1*. *P < 0.05, compared with non-transfected cells by Student’s *t* test

We next assessed whether *MUC1* positively regulated the *TFF2* expression in two GC cell lines (BGC-823 and SGC-7901). The *TFF2* expression level was measured by qRT-PCR after GC cell lines transfected with *MUC1* overexpression vector or siRNA. The results showed that overexpression of *MUC1* in BGC-823 and SGC-7901 cell lines substantially increased the *TFF2* expression, whereas inhibition of *MUC1* reverted this effect (Fig. 3).

### Overexpression of *MUC1* promotes hypomethylation of *TFF2*

**Fig. 4:**
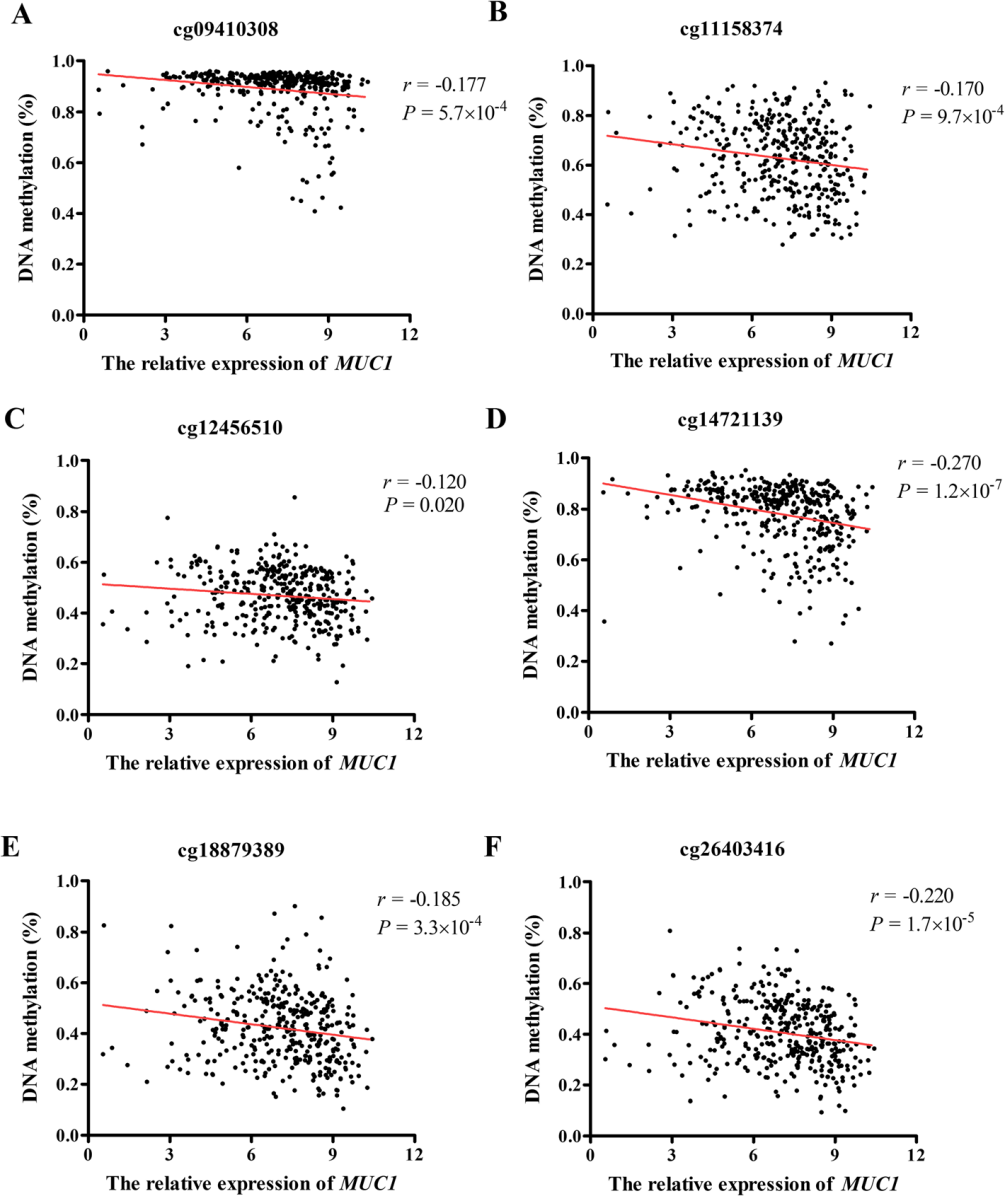
Correlations between *MUC1* expression and methylation level of CpG loci located in *TFF2*

We investigated whether the methylation levels of *MUC1* was associated with the dysregulation of *MUC1*. The results indicated that seven CpG sites were negatively associated with the *MUC1* expression (Supplementary Table 3). Given that high *MUC1* expression is correlated with increased the *TFF2* level, we then evaluated the associations of methylation level of *TFF2* and *MUC1* expression. The detail description of CpG sites located in *TFF2* gene is summarized in Supplementary Figure 4. As presented in Table 1 and Fig. 4, significant associations between *MUC1* expression and methylation levels of cg14721139, cg11158374, cg12456510, cg26403416, cg18879389, and cg09410308 were identified in GC tumor sample (all *P* < 0.05). Furthermore, we found that the expression level of *TFF2* was negatively related to the methylation status of these six CpG sites (all *P* < 0.05; Supplementary Figure 5). Subsequently, we conducted the correlation analysis of these six CpG sites themselves, and the results showed that the methylation level of these six CpG between each other were all prominently positive correlated (Supplementary Figure 6).

**Table 1:** Correlation analysis of methylation levels of *TFF2* with *MUC1* expression in gastric tumor tissues

CpG site	Position	Gene region	Coefficient	*P*^a^
cg14721139	Chr21:43772498	Promoter	−0.270	1.18E − 07
cg12234272	Chr21:43772018	Promoter	0.026	0.622
cg00355325	Chr21:43771923	Promoter	0.032	0.540
cg11158374	Chr21:43771664	Promoter	−0.170	9.72E − 04
cg12456510	Chr21:43771356	Promoter	−0.120	1.99E − 02
cg26403416	Chr21:43771340	Promoter	−0.220	1.73E − 05
cg18879389	Chr21:43771121	Promoter	−0.185	3.31E − 04
cg00083685	Chr21:43767586	Gene body	−0.084	0.106
cg09410308	Chr21:43766606	Gene body	−0.177	5.73E − 04

^a^*P* value for Pearson’s correlation analysis

To better understand of the regulatory mechanism, BSP was performed to compare the methylation level in GC cell lines transfected with negative vector or *MUC1* overexpression vector. In line with the correlation analysis, BSP results revealed that overexpression of *MUC1* contributed to a remarkable decrease in methylation of CpG site located in *TFF2* in BGC-823 cells (*P* = 0.041), and similar reduce trend was observed in SCG-7901 cell line (*P* = 0.078) (Table 2).

**Table 2:** Difference of *TFF2* methylation level between control and *MUC1* overexpression group

Variables	cg14721139 (%)	cg11158374 (%)	cg12456510 (%)	cg26403416 (%)	cg18879389 (%)	cg09410308 (%)	*P*^a^
BGC-NC	70	55	25	20	10	95	
BGC-OE	25	55	0	10	5	65	0.041
SGC-NC	70	90	15	10	25	95	
SGC-OE	40	80	15	5	0	95	0.078

^a^*P* value for paired *t* test

We also detected the effect of *MUC1* on expression of DNA methylation transferases (DNMTs), including *DNMT1*, *DNMT3a*, and *DNMT3b*. The results indicated that both overexpression and inhibition of *MUC1* leaded to a slight increase trend of DNMTs, especial *DNMT3a* (Supplementary Figure 7). These results suggested that *MUC1* was not associated with DNMTs expression.

### Downregulation of *MUC1* and *TFF2* are associated with poor outcome of GC

**Fig. 5:**
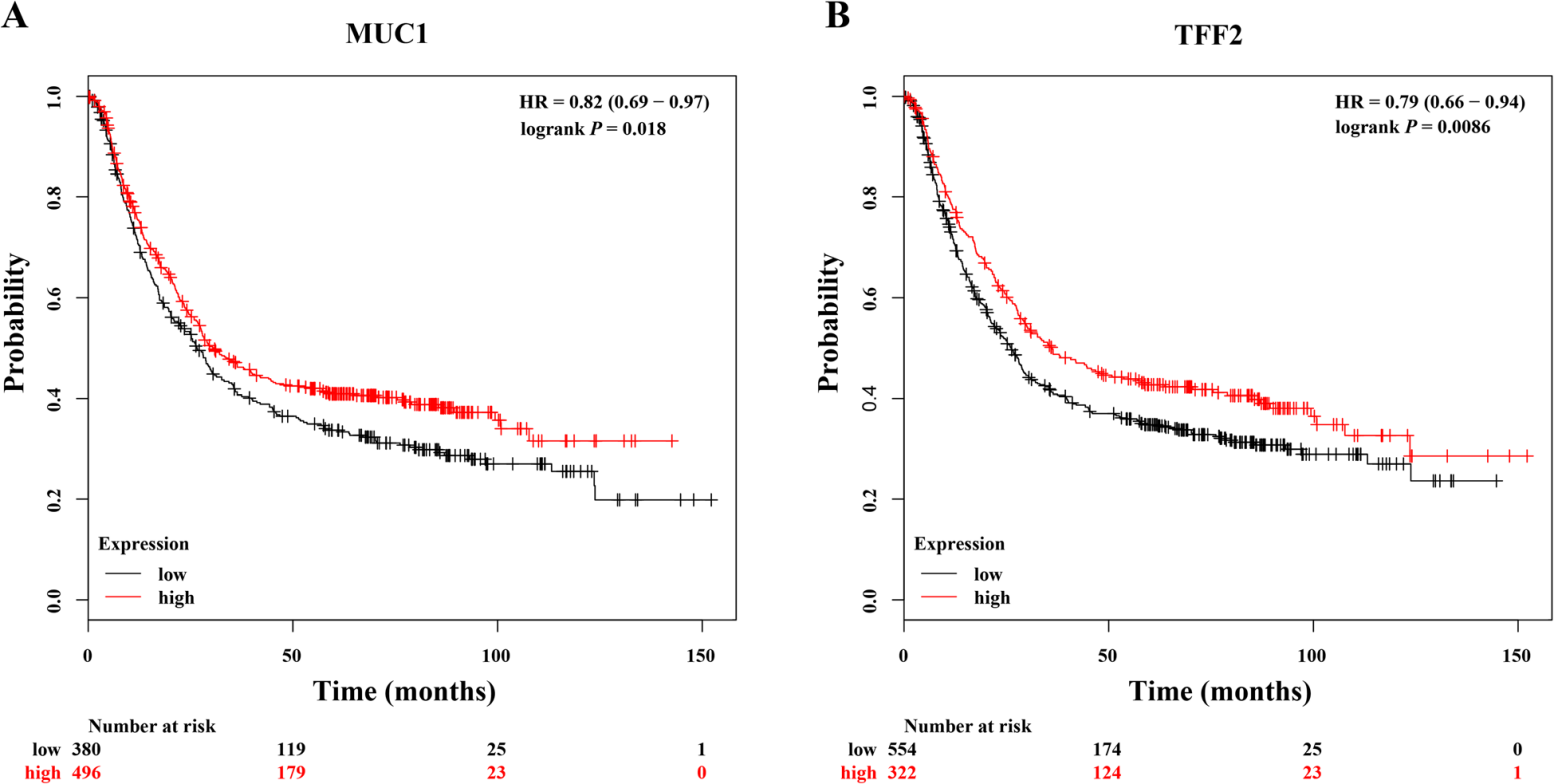
Kaplan-Meier curve of overall survival in GC patients according to *MUC1* and *TFF2* expression by Kaplan Meier plotter

We further investigated the prognosis effect of *MUC1* and *TFF2* on survival of GC patients by utilizing an online tool, Kaplan-Meier Plotter, which integrated gene expression data and survival information of GC patients. Prominent associations of *MUC1* and *TFF2* with outcomes of GC patients were obtained. The Kaplan-Meier plot showed that GC patients with high expression of *MUC1* had better outcome, with HR of 0.82 (95% CI, 0.69–0.97) comparing those with low level of *MUC1* (log-rank *P* = 0.018; Fig. 5a). In addition, higher *TFF2* expression was also obviously related to lengthen overall survival of GC patients (HR = 0.79, log-rank *P* = 0.009; Fig. 5b).

Furthermore, we divided GC patients into four groups according to the median of *MUC1* and *TFF2* expression by using TCGA database, including *MUC1* low+*TFF2* low, *MUC1* low+*TFF2* high, *MUC1* high+*TFF2* low, and *MUC1* high+*TFF2* high. Kaplan-Meier curve showed no significant difference of overall survival between four groups (Supplementary Figure 8). The result of multivariate cox regression analysis is summarized in Supplementary Table 4. After adjustment for age, sex, grade, and TNM stage, the *MUC1* and *TFF2* expression were not related to survival of GC patients.

## Discussion

There has been wide consensus that both mucins and TFF peptides play crucial roles in protecting mucosal epithelial cells from a variety of insults [21, 22]. In the present study, we demonstrated that the expression of *MUC1* and *TFF2* were significantly lower in GC tissues than that in adjacent normal tissues. *MUC1* positively regulated the expression of *TFF2* through correlation and experimental analysis. In addition, we observed that the methylation status of *TFF2* was prominent related to the *MUC1* expression. Furthermore, consistent with co-expression analysis findings, negative regulation of *MUC1* on methylation level of *TFF2* was also revealed in our BSP experiment, suggesting the role of *MUC1* in mediating *TFF2* expression by decreasing the methylation level of *TFF2*.

Epidemiology studies have demonstrated that about 50% of human infects *H. pylori* over the world and severe gastric disease occurs in 10–15% of infected carriers [23, 24]. Host factors play a key role in determining the susceptibility of *H. pylori*-infected carriers developed into GC. Mounting evidence shows that cell surface mucins protect gastric epithelial cell by their defense against *H. pylori* infection [25]. There are multiple members of mucins expressed in a variety of mucosal tissues. Among them, *MUC1* is the most widely investigated mucin and is identified to be abundantly expressed in all mucosal tissues [25]. Many studies have reported that the extracellular domain of *MUC1* is hypervariable and contains a variety of tandemly repeated 20-amino acid unites, which is well known to be associated with the increased risk of GC [26–28]. Previous GWASs have suggested significant associations between the *MUC1* risk loci and susceptibility of GC [12, 13]. SNP rs4072037 was reported to be pathogenic in GC for it altered the alternative splicing and transcriptional regulation of *MUC1* [29, 30]. Indeed, our results confirmed that *MUC1* was significantly lower in whether cardia or non-cardia tumor tissues than that in adjacent normal tissues. All findings described above highlight the key role of *MUC1* in the carcinogenesis of GC.

Epigenetic aberrations also play a key role in the pathogenesis of GC. It is generally agreed upon that *H. pylori* infection induced extensive DNA methylation alterations in gastric epithelial cells [31]. Numerous studies have shown that dysregulated promoter methylation contributed to silencing of tumor suppressor genes [32, 33]. A recent study revealed that *H. pylori* infection increased methylation level and decreased expression of *TFF2*, thus implicating in the development of GC [16]. TFF is a member of secreted protein family, which is composed by *TFF1*, *TFF2*, and *TFF3*, and characterized by the trefoil domain [34]. A lot of published studies revealed that the *TFFs* are involved in multiple biological processes, including barrier protection, cell proliferation, and apoptosis [35–37]. *TFF1* is a well-known gastric-related tumor suppressor gene, whereas the function of *TFF2* in GC is relatively less investigated. Accumulating data has shown that *TFF2* expression is frequently silenced in GC. In this study, we also found an obvious lower expression level of *TFF2* in GC tissues than that in adjacent normal tissue. Besides, previous experiments indicated that *Tff2*^−/−^ mice promoted the progression of gastritis to dysplasia after *H. pylori* infection [38]. Another study revealed that *TFF2* reduced proliferation of pancreatic ductal adenocarcinoma [39]. Additionally, the tumor suppressor effect of *TFF2* was also found in prostate and breast cancer [40, 41].

There has been wide consensus that epithelial cells synthesize and secrete TFF peptides together with mucins [42]. Intriguingly, *Muc1*^−/−^ mice infected with *H. pylori* obtained the similar phenotypes that appeared in *Tff2*^−/−^ mice. Furthermore, comparing with the infected controls, the infected *Muc1*^−/−^ mice prominently increased methylation level of *TFF2* [17]. Nevertheless, limited study is available to reveal the regulation mechanism between *MUC1* and *TFF2*. Our results showed that significantly positive associations between *MUC1* and *TFF2* were observed in tumor tissues and GC cell lines. Yamaguchi et al. found that *TFF2* was downregulated by promoting methylation of its promoter in pancreatic ductal adenocarcinoma, which was also identified in *H. pylori*-related GC [16, 39]. Considering that aberrant methylation of *TFF2* were involved in the carcinogenesis of tumor, we carried out co-expression analysis between *MUC1* and methylation of CpG loci located in *TFF2*. The results showed that the expression level of *MUC1* was reversely related to the methylation status of *TFF2*. It has been established that epigenetic dysregulation in promoter of genes plays an important role in the development of GC [43, 44]. Intriguingly, majority of these significant CpG loci are located in the promoter region of *TFF2*. Bioinformatic analysis indicated that transcription factors MAX, TCF12, and EZH2 may bind with the promoter of *TFF2* (https://www.genecards.org/). Consistent with findings in the earlier study, negative associations between *TFF2* methylation and the expression of *TFF2* were also detected in our correlation analysis. Moreover, BSP experiments further confirmed that overexpression of *MUC1* decreased the *TFF2* methylation. These results suggested the negatively regulate effect of *MUC1* on methylation level of *TFF2*, contributing to decrease *TFF2* levels. Given the important role of *MUC1* and *TFF2* in the development of GC, we have also investigated their prognosis effect. Our results indicated that GC patients with low level of *MUC1* or *TFF2* had poorer outcome, indicating that *MUC1* and *TFF2* may act as prognostic biomarkers in GC.

Some limitations are worthy of attention in the present study. Firstly, because numerous studies have supported the role of mucins and TFF peptide in the *H. pylori*-related pathologies, we investigated the regulation mechanism of *MUC1* implicated in the expression of *TFF2* without consideration of the *H. pylori* infection. In addition, we assessed co-expression between the *MUC1* and *TFF2* in public databases and our samples. Although the *P* value for correlation analysis was statistically significant, the coefficient (*r*) between *MUC1* and *TFF2* expression was relatively small. Moreover, this study found that *MUC1* could regulate the DNMTs expression slightly, suggesting *MUC1* was not associated with the expression of DNMTs. The underlying mechanism of *MUC1* influenced the methylation status of *TFF2* need to be further explored. Besides, whether *MUC1* and *TFF2* could act as the independent prognostic factors for GC remains to be investigated. The clinical studies of multicenter and larger samples are needed to validate our findings.

In conclusion, by using integrative analysis of the expression profiles and molecular experiments, we revealed that *MUC1* was negatively associated with the methylation of *TFF2* and positively regulated *TFF2* expression in GC. These findings provided new light on the strong link between *MUC1* and *TFF2* regulation. Further studies are warrant to explore the underlying epigenetic mechanism of this relationship in the pathogenesis of GC.

## Supplementary information

**Additional file 1: Table S1.** The primers for amplification in BSP experiments.

**Additional file 2: Table S2.** Subgroup analysis of the correlation of *MUC1* and *TFF2* expression in TCGA database.

**Additional file 3: Table S3.** Correlation analysis of *MUC1* methylation levels with *MUC1* expression in GC tissues.

**Additional file 4: Table S4.** Multivariate cox regression analysis for survival of GC patients in TCGA database.

**Additional file 5: Figure S1.** The expression of *MUC1* (A) and *TFF2* (B) in non-cardia and cardia tissue from GSE29272.

**Additional file 6: Figure S2.***H. pylori* infection and the expression of *MUC1* and *TFF2*. (A-B) The *MUC1* and *TFF2* expression in adjacent GC tissues with or without *H. pylori* infection from our cohort. (C-D) The expression change of *MUC1* and *TFF2* in GES-1 cells after 24 h-infection with *H. pylori* (GSE74577).

**Additional file 7: Figure S3.** The difference of methylation levels of CpG sites located in *MUC1* in GC patients with or without *H. pylori* infection (GSE99553 and TCGA). *P < 0.05; ** *P* < 0.01; *** *P* < 0.001.

**Additional file 8: Figure S4.** A schematic representation of the 9 CpG sites located in *TFF2* gene.

**Additional file 9: Figure S5.** Correlations between *TFF2* expression and methylation level of CpG loci located in *TFF2*.

**Additional file 10: Figure S6.** The Correlation analysis of the significant CpG sites located in *TFF2*. The values showed the Pearson’s correlation coefficient and corresponding 95% confidence interval.

**Additional file 11: Figure S7.** The normalized mRNA expression of *DNMT1*, *DNMT3a* and *DNMT3b* in BGC-823 and SGC-7901 cell line with *MUC1* overexpression (A) or inhibition (B).

**Additional file 12: Figure S8.** Kaplan Meier curve for overall survival of GC patients in four groups: *MUC1* low+*TFF2* low; *MUC1* low+*TFF2* high; *MUC1* high+*TFF2* low; *MUC1* high+*TFF2* high from TCGA database.

## Data Availability

The Cancer Genome Atlas (TCGA) database was available by the UCSC Xena project (http://xena.ucsc.edu/). The Gene Expression Omnibus (GEO) was publicly available online at https://www.ncbi.nlm.nih.gov/geo/, and the accession numbers are GSE29272, GSE74577, and GSE99553.

## Abbreviations

95% CI: 95% confidence interval
BSP: Bisulfite sequencing PCR
DNMTs: DNA methylation transferases
GC: Gastric cancer
GEO: Gene Expression Omnibus
GWASs: Genome-wide association studies
HR: Hazard ratio
*MUC1*: Mucin 1
NC: Negative control
qRT-PCR: Quantitative real-time PCR
siRNA: Small interfering RNA
TCGA: The Cancer Genome Atlas
*TFF2*: Trefoil factor family 2
