# Supplementary material for: MUC1 is associated with TFF2 methylation in gastric cancer
Source: Clin Epigenetics. 2020 Mar 2;12:37. doi: 10.1186/s13148-020-00832-6 (PMC7053135; doi:10.1186/s13148-020-00832-6)
Supplement: Supplementary file 1 — Additional file 1: Table S1. The primers for amplification in BSP experiments. [file 13148_2020_832_MOESM1_ESM.docx]

**Supplementary Table 1.** The primers for amplification in BSP experiments

| Primers | Sequence (5‘-3’) |
| --- | --- |
| F1 | AGTTTTTGTGGTTGTTTAGTGGTG |
| R1 | AACCCCTATCCTCCAAACTCTTAT |
| F2 | TTGGTTGAAAATGAATTTATTAAAATTT |
| R2 | TCAACTACACCCCAAAATAACTTAC |
| F3 | GAAGTTTTTGGGTAAAGTGATGTA |
| R3 | TAAAAAAACCCAAAATTTCATAAAATATAA |
