# Supplementary material for: MUC1 is associated with TFF2 methylation in gastric cancer
Source: Clin Epigenetics. 2020 Mar 2;12:37. doi: 10.1186/s13148-020-00832-6 (PMC7053135; doi:10.1186/s13148-020-00832-6)
Supplement: Supplementary file 2 — Additional file 2: Table S2. Subgroup analysis of the correlation of MUC1 and TFF2 expression in TCGA database. [file 13148_2020_832_MOESM2_ESM.docx]

**Supplementary Table 2.** Subgroup analysis of the correlation of *MUC1* and *TFF2* expression in TCGA database

|  | Coefficient | *P*^a^ |
| --- | --- | --- |
| Age (year) |  |  |
| ≤60 | 0.454 | <0.001 |
| >60 | 0.441 | <0.001 |
| Sex |  |  |
| Male | 0.443 | <0.001 |
| Female | 0.465 | <0.001 |
| Stage |  |  |
| I/II | 0.404 | <0.001 |
| III/IV | 0.479 | <0.001 |
| Grade |  |  |
| I/II | 0.505 | <0.001 |
| III | 0.386 | <0.001 |

^a^*P* value for Pearson’s correlation analysis
