# Supplementary material for: MUC1 is associated with TFF2 methylation in gastric cancer
Source: Clin Epigenetics. 2020 Mar 2;12:37. doi: 10.1186/s13148-020-00832-6 (PMC7053135; doi:10.1186/s13148-020-00832-6)
Supplement: Supplementary file 3 — Additional file 3: Table S3. Correlation analysis of MUC1 methylation levels with MUC1 expression in GC tissues. [file 13148_2020_832_MOESM3_ESM.docx]

**Supplementary Table 3.** Correlation analysis of *MUC1* methylation levels with *MUC1* expression in GC tissues

| CpG sites | Position | Gene region | Coeffcient | *P*^a^ |
| --- | --- | --- | --- | --- |
| cg03055449 | chr1:155163662 | promoter | 0.033 | 0.528 |
| cg06420088 | chr1:155163590 | promoter | 0.013 | 0.800 |
| cg17257175 | chr1:155163543 | promoter | -0.004 | 0.944 |
| cg19755544 | chr1:155163240 | promoter | 0.012 | 0.815 |
| cg23256951 | chr1:155163206 | promoter | 0.0004 | 0.994 |
| cg07399355 | chr1:155163014 | promoter | 0.017 | 0.739 |
| cg02386822 | chr1:155162872 | promoter | -0.128 | 1.33E-02 |
| cg22531371 | chr1:155162756 | promoter | -0.249 | 1.15E-06 |
| cg22500132 | chr1:155162752 | promoter | -0.238 | 3.31E-06 |
| cg18804777 | chr1:155161834 | gene body | -0.533 | <0.001 |
| cg24512973 | chr1:155161785 | gene body | -0.480 | <0.001 |
| cg20949223 | chr1:155161680 | gene body | -0.593 | <0.001 |
| cg00930306 | chr1:155161222 | gene body | NA | NA |
| cg15699386 | chr1:155161212 | gene body | NA | NA |
| cg06216400 | chr1:155161162 | gene body | NA | NA |
| cg13804478 | chr1:155158319 | gene body | -0.306 | 1.65E-09 |

^a^*P* value for Pearson’s correlation analysis
