# Supplementary material for: MUC1 is associated with TFF2 methylation in gastric cancer
Source: Clin Epigenetics. 2020 Mar 2;12:37. doi: 10.1186/s13148-020-00832-6 (PMC7053135; doi:10.1186/s13148-020-00832-6)
Supplement: Supplementary file 4 — Additional file 4: Table S4. Multivariate cox regression analysis for survival of GC patients in TCGA database. [file 13148_2020_832_MOESM4_ESM.docx]

**Supplementary Table 4.** Multivariate cox regression analysis for survival of GC patients in TCGA database

| Variables | HR | 95%CI | *P* value |
| --- | --- | --- | --- |
| Age (≤60 *vs.* >60 year) | 1.94 | 1.31-2.86 | 0.001 |
| Sex (male *vs.* female) | 0.76 | 0.52-1.10 | 0.150 |
| Grade (I/II *vs.* III) | 1.49 | 1.04-2.14 | 0.032 |
| TNM (I/II *vs.* III/IV)) | 1.85 | 1.29-2.66 | 0.001 |
| MUC1 (low *vs.* high) | 0.84 | 0.59-1.19 | 0.320 |
| TFF2 (low *vs.* high) | 1.29 | 0.91-1.82 | 0.158 |
