# Supplementary figures and images for: MUC1 is associated with TFF2 methylation in gastric cancer
Source: Clin Epigenetics. 2020 Mar 2;12:37. doi: 10.1186/s13148-020-00832-6 (PMC7053135; doi:10.1186/s13148-020-00832-6)

**Supplementary Figure 1.**


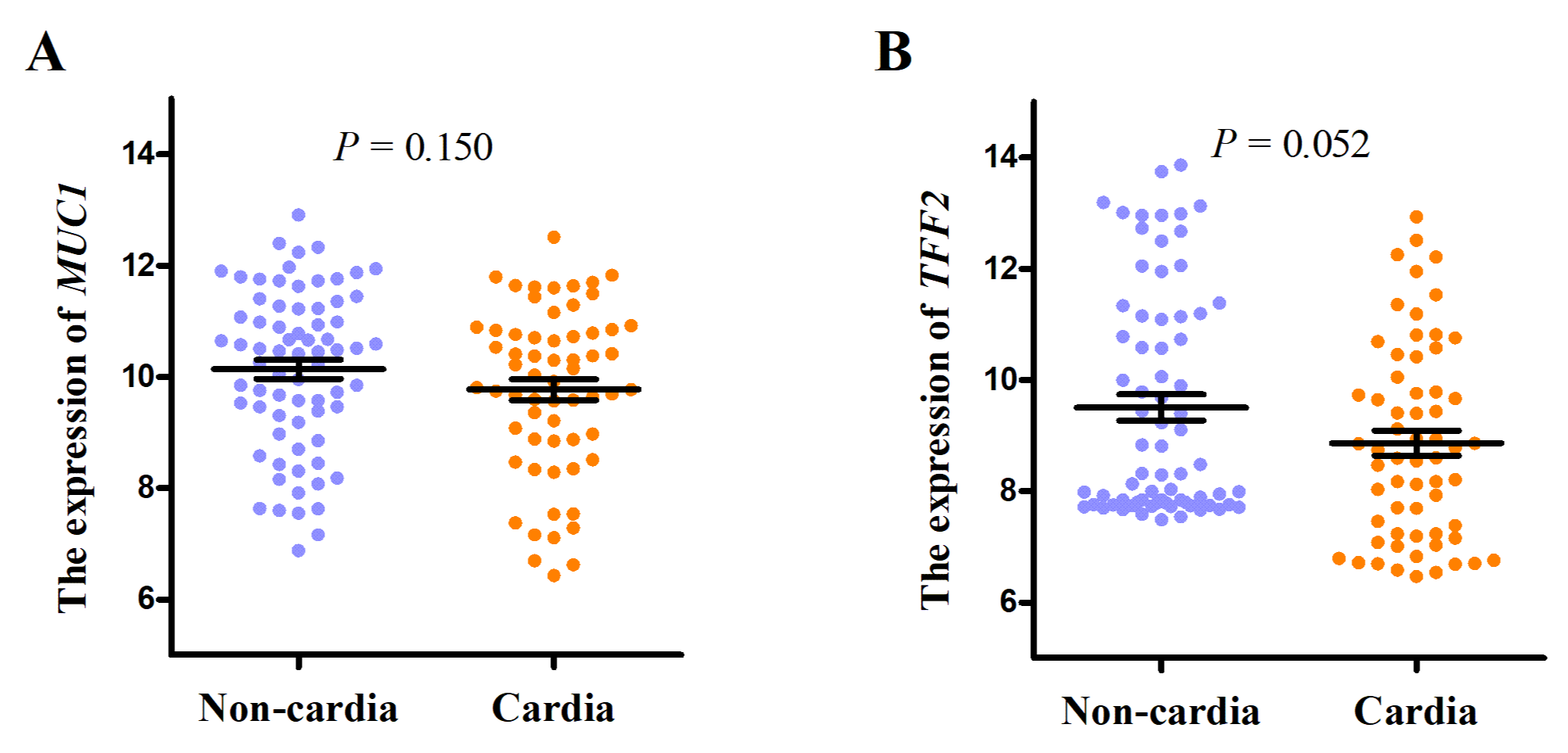

Supplement: Supplementary file 5 — Additional file 5: Figure S1. The expression of MUC1 (A) and TFF2 (B) in non-cardia and cardia tissue from GSE29272. [file 13148_2020_832_MOESM5_ESM.docx]

**Supplementary Figure 2.**


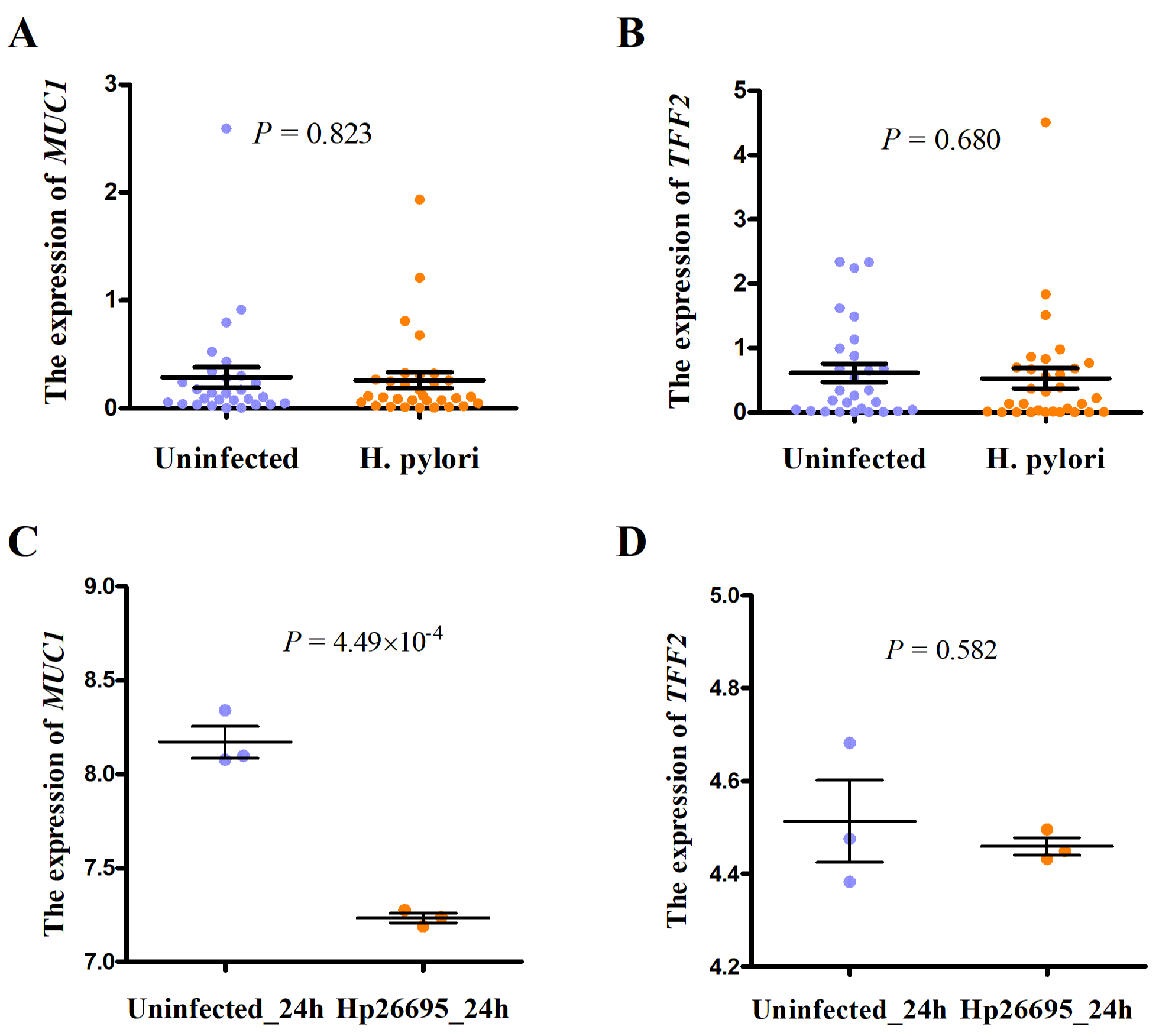

Supplement: Supplementary file 6 — Additional file 6: Figure S2.H. pylori infection and the expression of MUC1 and TFF2. (A-B) The MUC1 and TFF2 expression in adjacent GC tissues with or without H. pylori infection from our cohort. (C-D) The expression change of MUC1 and TFF2 in GES-1 cells after 24 h-infection with H. pylori (GSE74577). [file 13148_2020_832_MOESM6_ESM.docx]

**Supplementary Figure 3.**


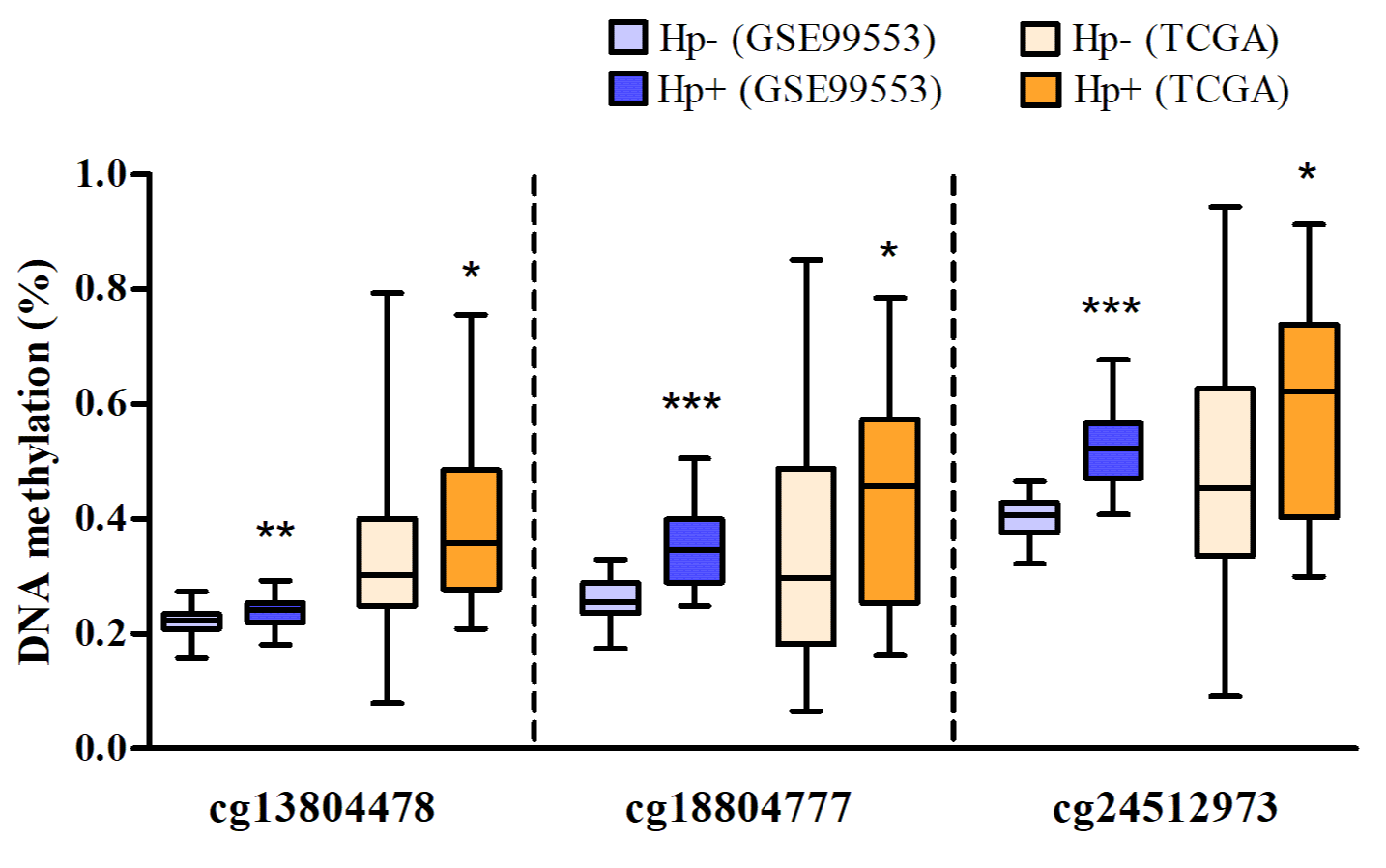

Supplement: Supplementary file 7 — Additional file 7: Figure S3. The difference of methylation levels of CpG sites located in MUC1 in GC patients with or without H. pylori infection (GSE99553 and TCGA). *P < 0.05; ** P < 0.01; *** P < 0.001. [file 13148_2020_832_MOESM7_ESM.docx]

**Supplementary Figure 4.**


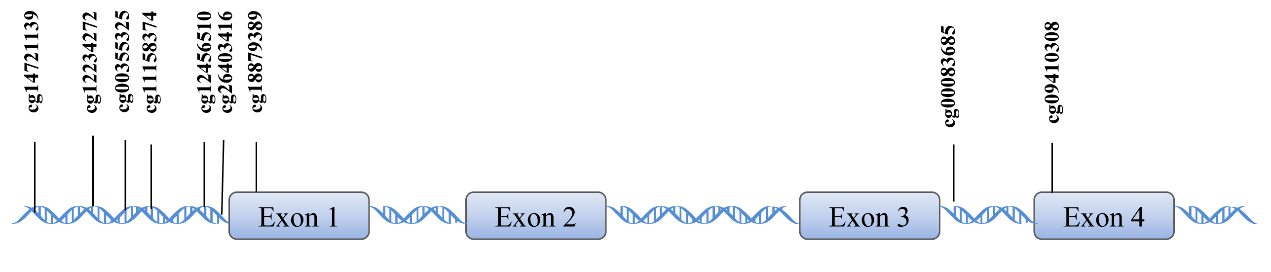

Supplement: Supplementary file 8 — Additional file 8: Figure S4. A schematic representation of the 9 CpG sites located in TFF2 gene. [file 13148_2020_832_MOESM8_ESM.docx]

**Supplementary Figure 5.**


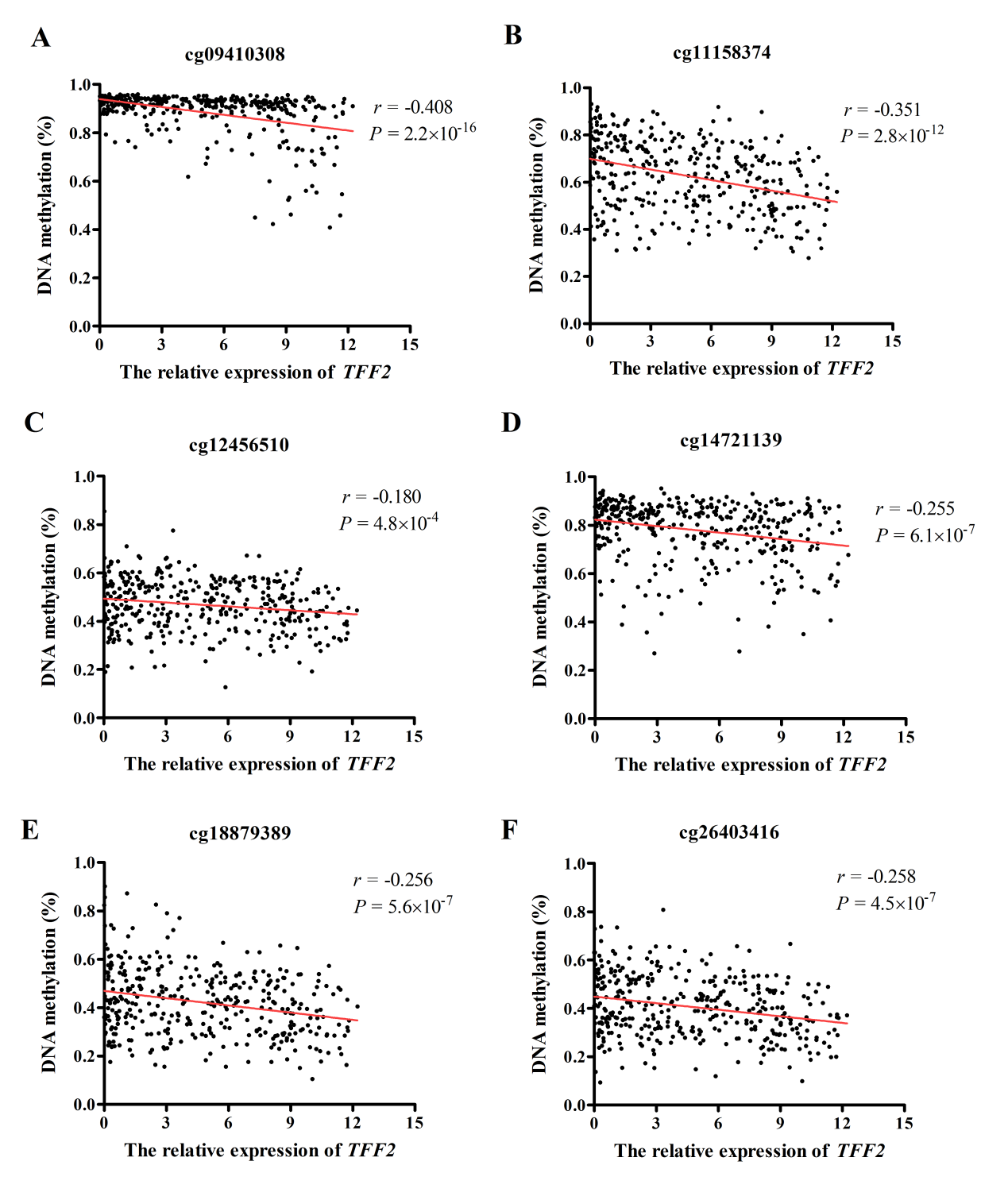

Supplement: Supplementary file 9 — Additional file 9: Figure S5. Correlations between TFF2 expression and methylation level of CpG loci located in TFF2. [file 13148_2020_832_MOESM9_ESM.docx]

**Supplementary Figure 6.**


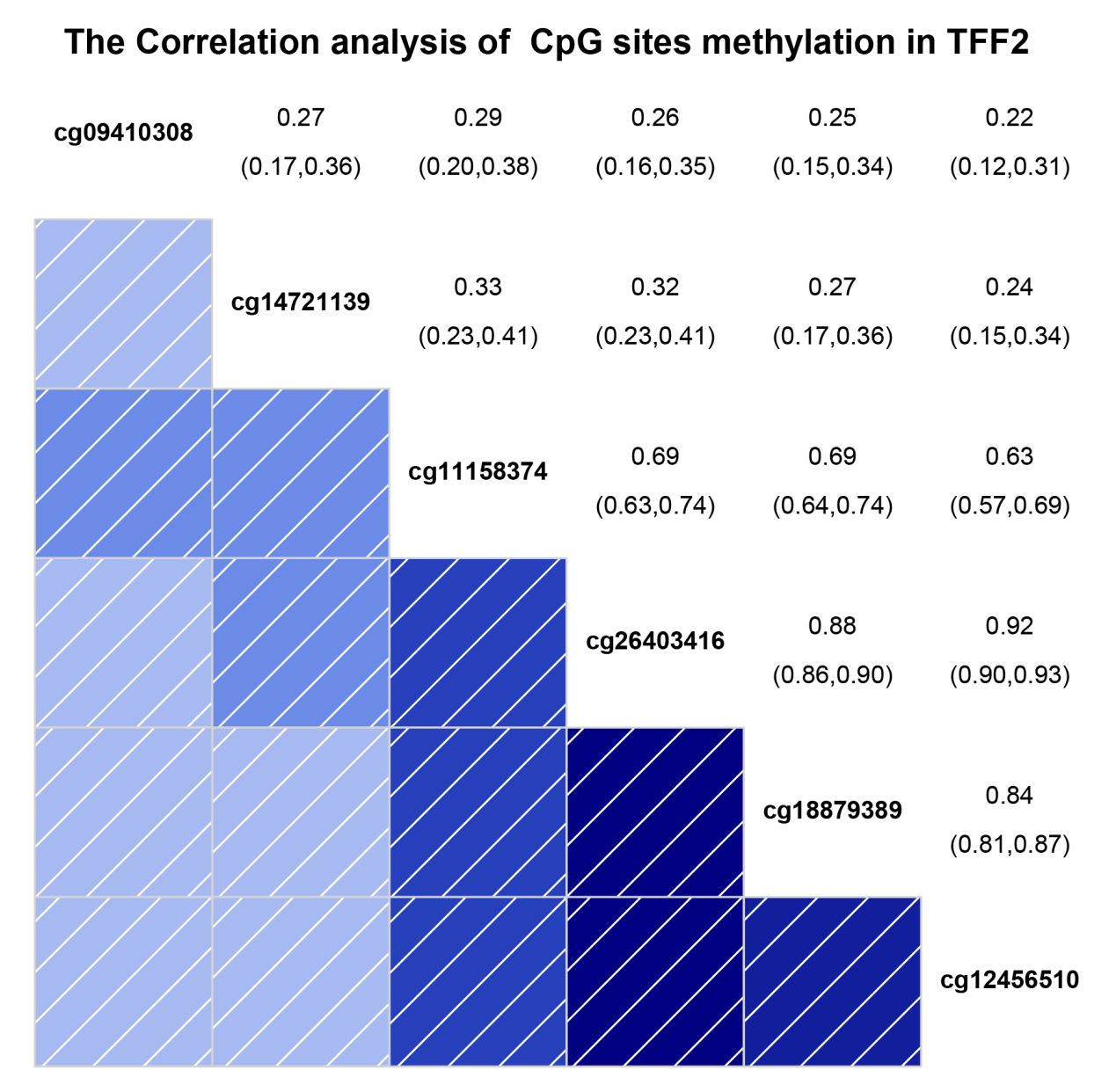

Supplement: Supplementary file 10 — Additional file 10: Figure S6. The Correlation analysis of the significant CpG sites located in TFF2. The values showed the Pearson’s correlation coefficient and corresponding 95% confidence interval. [file 13148_2020_832_MOESM10_ESM.docx]

**Supplementary Figure 7.**


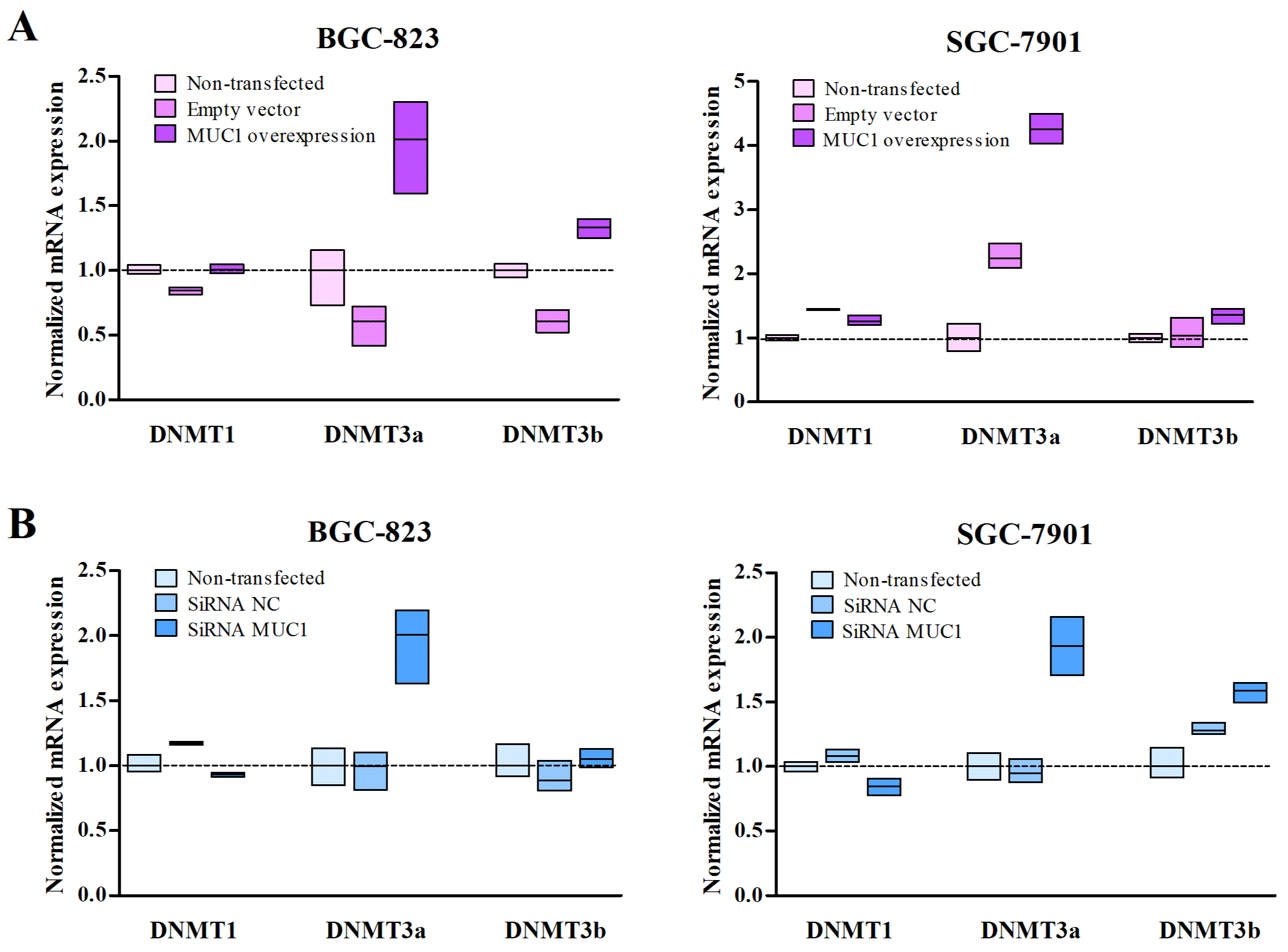

Supplement: Supplementary file 11 — Additional file 11: Figure S7. The normalized mRNA expression of DNMT1, DNMT3a and DNMT3b in BGC-823 and SGC-7901 cell line with MUC1 overexpression (A) or inhibition (B). [file 13148_2020_832_MOESM11_ESM.docx]

**Supplementary Figure 8.**


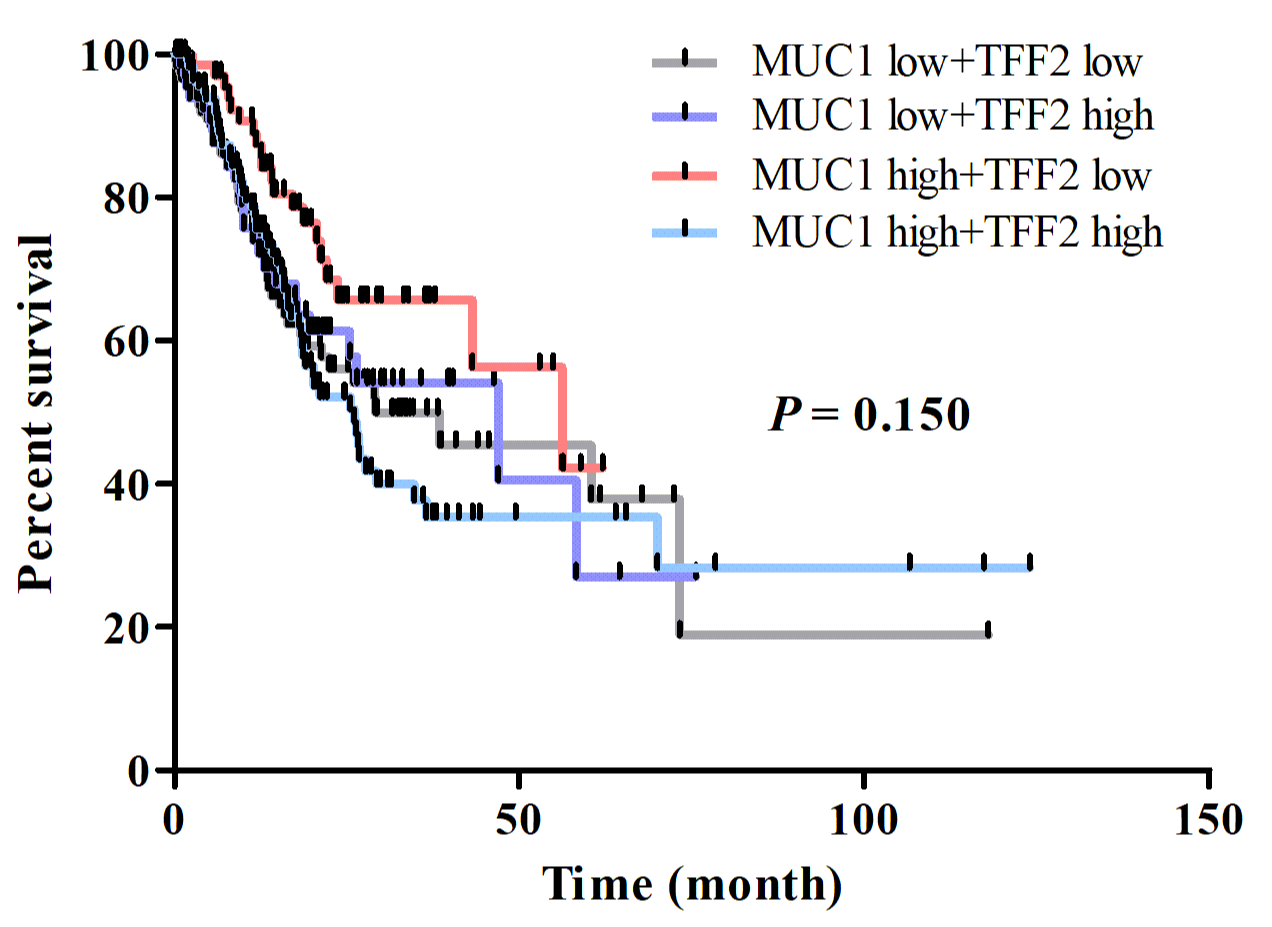

Supplement: Supplementary file 12 — Additional file 12: Figure S8. Kaplan Meier curve for overall survival of GC patients in four groups: MUC1 low+TFF2 low; MUC1 low+TFF2 high; MUC1 high+TFF2 low; MUC1 high+TFF2 high from TCGA database. [file 13148_2020_832_MOESM12_ESM.docx]
